# Supplementary material for: Patterns of Intron Gain and Loss in Fungi
Source: PLoS Biol. 2004 Nov 30;2(12):e422. doi: 10.1371/journal.pbio.0020422 (PMC532390; doi:10.1371/journal.pbio.0020422)
Supplement: Table S1 — Also available at http://genes.mit.edu/NielsenEtAl/. (4.3 MB ZIP). [file pbio.0020422.st001.zip › NielsenEtAl/html/293.html]

AN1115.1.NCU03084.1.MG04540.1.FG00465.1


```
 CLUSTAL W (1.82) Multiple Sequence Alignments - Introns Inserted


Sequence 1: NCU03084.1	438 aa
Sequence 2: FG00465.1	425 aa
Sequence 3: MG04540.1	433 aa
Sequence 4: AN1115.1	421 aa
Alignment Length: 444 aa
Number Identitical Residues: 165 aa
Alignment Score (without introns) 9360


MG04540.1 	MAPKHRIIIDTDP~G1VDDVLA~ILLALSASPEDLEVAMISVTYGNVPLQG2CLRNAVAL
NCU03084.1	MAPKQRVILDTDP~G~VDDVLA~LLLALSASPEELELAMISVTYGNVPLQS2CLRNVVAT
FG00465.1 	MAPKQKIILDTDP~-~-----A1LLLALSAKPEELEVLLVSVTYGNVPLQS2CLRNVVSA
AN1115.1  	MAPK-KIIIDTDP0G~IDDILA~LLLAFSQKAEDVEVLLVSLTFGNVEVKS2CLRNVVSM
          	**** ::*:**** .  ..  * :***:* ..*::*: ::*:*:*** ::. ****.*: 

MG04540.1 	FHVLEKERAWRKSVGKET-GFESLSVFKPIVAIGAKHPLEEEELAADHFH1GADGLHGVH
NCU03084.1	FHVLEKEMKWREETGKSTRGFGALNTHKPIVAVGPEHALEDEILAADHFH1GADGLHGVH
FG00465.1 	FHVIEKELEWRKSTGKPE-GFGAMKANKPIVAVGPDHPLCDEELMADYFH1GIDGLHNVH
AN1115.1  	FHILEREMKWRHENGKPE-GFDALRAHPPIVALGAEDPLDDQKMLADYFH1GIDGLGGIH
          	**::*:*  **.. **   ** :: .  ****:*....* :: : **:** * *** .:*

MG04540.1 	DA0HPDLTPAETWRTLFED-APGGAPSDIPS---FSTYFTPSKTLAHKEILRILKESPDQ
NCU03084.1	EK0QPHLSPADTWRSLFSDEEPQGEKVEPPS---YSKYFTPSKAPAHKEILRVLKESPEN
FG00465.1 	EA~HPDLSPADTWKGIFGDGAEGGE---------HSPLFTPSKAPSHQEILRILKENPAD
AN1115.1  	GS0HPHLTPQDAWEHLFDPTHEPVELEPVPSGSGAHRSFIPSKRPAHEEMLRILRENDPD
          	   :*.*:* ::*. :*            .:.:.    * ***  :*:*:**:*:*.  :

MG04540.1 	TVTILTIGPMTNLALAAAEDPETLLRAREVCVMGGAINVPGN~ITPVAEFNTFADAVATA
NCU03084.1	TVTVVAVGPLTNVALAAAEDPETFLRAKELVVMGGAVNVEGN~VTPVAEFNCFADTVAAA
FG00465.1 	TVSILAVGPLTNVALAAAEDPETFLRAKELVVMGGAVNVEGN~CTPVAEFNCYADAVAAA
AN1115.1  	TVTIVAVGPLTNLAVASAADPEAFLRAKEVVVMGGAVHEPGN0VTPVAEFNAYADAFAAA
          	**:::::**:**:*:*:* ***::***:*: *****::  **  ******* :**:.*:*

MG04540.1 	RVFALTAREPASTMPPCTS--PSLKPYPEGLSRQLKLSLFPLDITTDHLLNKGFFTERIA
NCU03084.1	RVYALTSPNPSSTMPPVIHGKSFLPPYPAKLSKQLKLTLFPLDITTPLELRKKYFYETIQ
FG00465.1 	RVYALTSLKPSSTMPIIPKELSTLKAYPEKLSRQLKLTLCPLDITTPHLIGKNYFKENIQ
AN1115.1  	RVYALTSPSPKSTLPFCKS----LPDLPPSLSKQLTVKIFPLDITSRHSINRGQFRKATA
          	**:***: .* **:*        *   *  **:**.:.: *****:   : :  * :   

MG04540.1 	PLVASGSPLARWLEHVLSRTFDKIDSILGD--GSEPGLSLHDPLTVWYMMTRDNPAWKAS
NCU03084.1	PITKSGSPLALWIETFMTGIFNKVEAMLGD--GSEPGLSLHDPACIWYMLTQDDPAWEPV
FG00465.1 	AHIDAGSPLARWVSHFVTASFKKIEEMEGD--ENEPGLSLHDPLTVWYMLTRDDPKWKTP
AN1115.1  	PLVEAGSPLAEWSTAFMAHTFATVERLHANQVGDAAELSLHDPVCVWYALTAEDEGWKPS
          	.   :***** *   .::  * .:: : .:.  . . ******  :** :* ::  *:. 

MG04540.1 	AG--EDIRVETAGQWTRGMHILDRRVRSKPGVKQEAGQAEKTEVVDGVQLDEVPGDTMGW
NCU03084.1	AKP-EDIRIETSGQWTRGMHVIDRRQRAKPGEQSSKVETHPSDPLDATTFDDVPGDDMGW
FG00465.1 	EKL-EDIRVETSGQWTHGMHVVDRRFRKKPAEAAVALSANPTEDPQILALDEVPGDDHAW
AN1115.1  	EKSPEDIRIETTGQWTRGACIIDRRDR-HPVEGE----------------EESSSDHGLW
          	   .****:**:****:*  ::*** * :*                    :: ..*   *

MG04540.1 	LSRRRGNSVNRIVGSPGFNIFAEYLMKQLFW
NCU03084.1	LSVNKGNRINRMVKTPGDEKFAAILMDRLFG
FG00465.1 	LSVLKGNRLNRVIDSPGHDIFKEVLMQRIFG
AN1115.1  	LSSKAGNRIWRMDESPVEDNFGTVLIEQLFI
          	**   ** : *:  :*  : *   *:.::*
```
